# Tumors Alter Inflammation and Impair Dermal Wound Healing in Female Mice

**DOI:** 10.1371/journal.pone.0161537

**Published:** 2016-08-22

**Authors:** Leah M. Pyter, Yasmin Husain, Humberto Calero, Daniel B. McKim, Hsin-Yun Lin, Jonathan P. Godbout, John F. Sheridan, Christopher G. Engeland, Phillip T. Marucha

**Affiliations:** 1 Institute for Behavioral Medicine Research, Ohio State University Wexner Medical Center, Columbus, OH, United States of America; 2 Department of Psychiatry and Behavioral Health, Ohio State University, Columbus, OH, United States of America; 3 Department of Neuroscience, Ohio State University, Columbus, OH, United States of America; 4 Center for Wound Healing and Tissue Regeneration, College of Dentistry, University of Illinois at Chicago, Chicago, IL, United States of America; 5 Deparment of Biosciences, College of Dentistry, Ohio State University, Columbus, OH, United States of America; 6 Department of Biobehavioral Health and College of Nursing, Pennsylvania State University, University Park, PA, United States of America; 7 College of Dentistry, Oregon Health and Sciences University, Portland, OR, United States of America; Cedars-Sinai Medical Center, UNITED STATES

## Abstract

Tissue repair is an integral component of cancer treatment (e.g., due to surgery, chemotherapy, radiation). Previous work has emphasized the immunosuppressive effects of tumors on adaptive immunity and has shown that surgery incites cancer metastases. However, the extent to which and how tumors may alter the clinically-relevant innate immune process of wound healing remains an untapped potential area of improvement for treatment, quality of life, and ultimately, mortality of cancer patients. In this study, 3.5 mm full-thickness dermal excisional wounds were placed on the dorsum of immunocompetent female mice with and without non-malignant flank AT-84 murine oral squamous cell carcinomas. Wound closure rate, inflammatory cell number and inflammatory signaling in wounds, and circulating myeloid cell concentrations were compared between tumor-bearing and tumor-free mice. Tumors delayed wound closure, suppressed inflammatory signaling, and altered myeloid cell trafficking in wounds. An *in vitro* scratch “wounding” assay of adult dermal fibroblasts treated with tumor cell-conditioned media supported the *in vivo* findings. This study demonstrates that tumors are sufficient to disrupt fundamental and clinically-relevant innate immune functions. The understanding of these underlying mechanisms provides potential for therapeutic interventions capable of improving the treatment of cancer while reducing morbidities and mortality.

## Introduction

Tissue repair is a fundamental and evolutionarily conserved immune process [1]. Certain chronic disease populations require extensive tissue repair as part of successful treatment of their disease. For example, cancer patients undergo at least one treatment procedure that damages healthy tissue: surgery, radiation, chemotherapy [2]. Given the systemic immune changes caused by cancer, it is likely that the clinically-relevant process of wound healing becomes compromised in these patients. Empirical data on wound healing during cancer are underwhelming [3, 4]. One study reports 34% more non-healing surgical wounds in cancer patients relative to cancer-free controls [5]. Extra time is allotted for proper stabilization of wounds in cancer patients in order to maximize successful reconstruction [6], supporting evidence that healing is impaired. However, the limited oncological data on wound healing is often secondary to other aims and lack cancer-free controls [7, 8]. Several older studies in rats also suggest that tumors reduce wound strength, possibly through cachexia-related mechanisms [9–11]. Impairments or delays in the healing of these wounds are particularly injurious because they can delay tumor removal and treatment progress [6], increase morbidity, disfigurement/scarring, and stress [12], and potentially cause secondary cancerous lesions to form [13].

The typically late diagnosis and diffuse nature of head and neck cancer (HNC), in particular, causes tissue-damaging treatments to be common, extensive, and numerous [12, 14]. The long duration of surgery time for these tumors also contributes to poor healing outcomes. Pre-treatment biopsy wounds, tooth extractions, portacath, tracheotomies, feeding tube insertionS, and other minor dental surgeries are common preventative procedures [15] that require proper healing and stabilization before surgical tumor resection can occur [16], thereby potentially extending the time for cancer growth and spread. HNC surgery causes high rates of infection (24–87%) [17–19], which is positively correlated with tumor stage [20] and morbidity [19]. Recurrence has stagnated at about 50% [14]. Thus, post-surgical healing often occurs in the presence of remaining cancer cells.

HNC is highly immunosuppressive [21, 22], characterized by elevations in bone marrow-derived circulating immature myeloid progenitor cells, often described as myeloid-derived suppressor cells (MDSCs). MDSCs have been studied primarily in terms of their suppressive effects on T-cell or natural killer cell number and function [23], although recent evidence suggests they also suppress macrophage inflammatory function [24]. Indeed, one study reports decreased macrophage fractions in surgical drainage fluid of cancer patients relative to non-cancer controls [25]. Normal wound healing consists of three overlapping and interdependent phases: hemostasis/inflammation, proliferation, and scar formation/remodeling. The inflammatory phase is characterized by chemotactic signaling (e.g., CCL2, CCL3, CXCL8) from resident keratinocytes, platelets, and parenchyma cells to recruit neutrophils and macrophages [26, 27]. Both of these cell types phagocytose invading microorganisms and debris and promote inflammation through cytokine release (e.g., IL-1β, IL-6, TNFα). Neutrophils are the first responders, with peak wound infiltration one day post-wounding, whereas macrophages peak around Day 3 post-wounding [28, 29]. Thus, through inhibitory effects on myeloid cells, cancer-induced immunosuppression may impair wound healing during this initial inflammatory phase.

For this work, we hypothesized that distal oral squamous cell carcinomas delay dermal wound healing in mice and are associated with altered inflammation. By better understanding the interactions between tumors and wounds, biomarkers can be used as a predictor of healing responses during cancer treatment and mechanism-specific interventions can be implemented. Improved healing would decrease morbidity and costs, accelerate cancer treatment plans, and ultimately reduce patient mortality. In addition to being of considerable clinical significance, this work contributes to the paucity of biological understanding of how essential immune functioning is affected during cancer.

## Materials and Methods

### Animals

Nulliparous female C3H mice (Charles River, Wilmington, MA) were housed 5/cage and acclimated to the vivarium for 1–2 weeks. Females were used to avoid non-experimental wounding from fighting that occurs in cohabitating males. Mice were housed using a 14:10 light:dark cycle with lights on at 06:00 h in a facility controlled for temperature (21 ± 1°C). Rodent chow (Harlan 7912) and water were available *ad libitum* throughout the study and shredded paper was available for nest building. All animal experiments were approved by the University of Illinois at Chicago or Ohio State University Institutional Animal Care and Use Committee and carried out in accordance with the National Institutes of Health Guide for the Care and Use of Laboratory Animals (Institute for Laboratory Animal Resources, 1996). All efforts were made to minimize animal suffering and to reduce the number of mice used.

### Cells

The murine oral cancer cell line AT-84, originating from C3H mice [30], was generously provided by Dr. Shulin Li at MD Anderson (Houston, TX, USA) and grown in RPMI-1640 with 10% FBS, 2 mM _L_-glutamine, 0.1 mM non-essential amino acids,10 mM N-2-HEPES buffer, 100 units penicillin/ml, and 100 μg streptomycin/ml at 37°C with 5% CO_2_.

### Cancer

Between 8–10 weeks of age, 100 mice were separated equally into cancer and control treatment groups. Mice were acclimated to handling three times in the preceding two weeks. They were anesthetized (100 mg/kg ketamine mixed with 10 mg/kg xylazine, i.p.) and then injected in the flank (s.c.) with either a 150 μl suspension of 1.5 x 10^6^ AT-84 cells (n = 50) or PBS vehicle (n = 50). Ear notches were made at this time for identification purposes. This procedure results in oral squamous cell carcinomas [30–32], the most common type of oral cancer in humans [33], that do not metastasize (Lou 2003). This is a validated syngeneic model, allowing for the immune system to remain intact. Body mass was measured twice/week until wounding, at which time body mass was measured daily.

### Dermal Wounding Procedure and Analysis

When average tumor dimensions were ~0.5 x 0.5 cm (16 d post-tumor induction), mice were wounded. All mice were anesthetized again (100 mg/kg ketamine mixed with 10 mg/kg xylazine, i.p.), the dorsal skin was shaved, cleaned with alcohol, and wounded by placing two full-thickness excisional wounds through the dorsal skin using 3.5 mm sterile biopsy punches (Miltex Instrument Company, Plainsboro, NJ, USA) just below the shoulder blades to prevent mice from licking their own wounds. Wound closure was assessed via daily pictures of the wounds (through Day 5 post-wounding) and images were analyzed by a single investigator (L.P or Y.H.). Photographs of the biopsy sites were taken with a 3.5 mm standard-sized dot placed beside the wound to control for variations in photograph angle and distance. Wound size was measured by Adobe Acrobat Pro software [34] and expressed as the ratio of the wound area to the dot measurement, then as a ratio to the original wound size on Day 0 [34]. Wounds from half of the mice in all treatment groups were harvested using sterile 6.0 mm punch biopsies (Miltex Instrument Company) on Day 1 and the other half on Day 5 post-wounding (n = 5-12/group) following deep anesthetization (ketamine/xylazine). Wounds were either flash frozen for genomic comparison of factors known to regulate wound healing using quantitative RT-PCR (qRT-PCR; n = 9-12/group) or processed for flow cytometric analyses (n = 2-3/group). Cardiac whole blood was collected in heparin-lined syringes for complete blood count (CBC) analysis of circulating myeloid cells by the Biological Resources Laboratory at UIC. Additional groups of unwounded tumor-bearing and control mice (n = 8-10/group) sampled at the same time relative to tumor induction were added for these blood tests.

### Quantitative RT-PCR

Total RNA was extracted from wound tissue as described in detail elsewhere [35]. RNA concentrations were measured and 260/280 ratios were determined (NanoDrop, DE, USA). Total RNA was reverse transcribed using SuperScript First-Strand kits (Invitrogen, NY, USA) according to the manufacturer’s protocol. RNase H (1 μl) was added to each sample and incubated at 37°C for 20 min and then 1 μl cDNA was diluted 1:5 with RNAse-free water and stored in -20°C for qRT-PCR. Sixteen genes of interest were chosen based on their role in wound macrophage recruitment (Ccl2 [Mcp-1], Ccl3 [Mip-1], Cx3cl1 [fractalkine]), neutrophil recruitment (Cxcl1 [Kc], Cxcl2 [Mip-2]), pro-inflammatory (IL-1β, Tnfα, IL-6), anti-inflammatory (Il-10, Tgf-β, Ccl1), classical macrophage activation (Cxcl10, Ccl5, Tlr4), and wound healing macrophage activation (Ccl22, Igf-1). Mouse TaqMan Gene Expression Assays were purchased from Applied Biosystems (Carlsbad, CA, USA) with probes labeled with 6-FAM and MGB (non-fluorescent quencher) at the 5’ and 3’ ends, respectively: *Il-1β* (Mm00434228_m1), *IL-6* (Mm00446190_m1), *Ccl3* (Mm00441259_g1), *Cxcl1 (*Mm04207460_m1), *Il-10* (Mm00439614_m1), *Tgfβ* (Mm01178820_m1), *Tlr4* (Mm00445273_m1), *Tnfα* (Mm00443260_g1), *Ccl2 (*Mm00445109_m1), *Cxcl2 (*Mm00436450_m1), *Cx3cl1* (Mm00436454_m1), *Cxcl10 (*Mm00445235_m1), *Ccl22 (*Mm00436439_m1), *Ccl5 (*Mm01302427_m1), *Ccl1 (*Mm00441236_m1), *Igf-1 (*Mm00439560_m1), *Gapdh* (Mm99999915_g1; labeled with VIC). The universal two-step RT-PCR cycling conditions used on the 7000 Sequence Detection System (Applied Biosystems) were: 50°C (2 min), 95°C (10 min), 40 cycles of 95°C (15 s) and 60°C (1 min). Relative gene expression of individual samples run in duplicate was calculated by the comparative C_T_ method (2^-ΔCT^). Genes of interest that were below detectability were considered zero if their endogenous control value was within the normal range.

### Flow cytometry

In a subset of mice (n = 5/cancer treatment), tumor tissue was collected on Day 1 or 5 post-wounding and processed for myeloid cell quantification using flow cytometry. Briefly, both wounds were collected from each mouse using a 6.5 mm biopsy punch immediately following CO_2_ asphyxiation and collected in an enzymatic digestion solution (3.3 mg/ml each of collagenase I, collagenase XI, hyaluronidase) until further processing [36]. Wounds were then minced and rotated at 37°C for 1 h. The digest was filtered through a 70-μm nylon cell strainer, rinsed, centrifuged, resuspended, and then the total number of cells was determined using a BD Coulter Particle Count and Size Analyzer (Beckman Coulter). Staining of cell surface antigens was performed as previously described [37, 38]. In brief, Fc receptors were blocked with anti-CD16/CD32 antibody (eBioscience, San Diego, CA, USA) and then cells were incubated with the appropriate conjugated antibodies (CD11b [M1/70], Ly6C [AL-21], Ly6G [1A8], Il-4Rα [mIL4R-M1],BD Biosciences, San Jose, CA, USA) for 1 h at 4°C. Cells were washed and then resuspended in FACS buffer for analysis. Nonspecific binding was assessed using isotype-matched antibodies. Antigen expression was determined using a Becton-Dickinson FACSCalibur four-color cytometer (BD Biosciences). Data were analyzed using FlowJo software (Tree Star, Ashland, OR, USA) and positive labeling for each antibody was determined based on isotype stained controls.

### *In vitro* “wound” scratch assay

This assay is used as a simplified system by which to study healing-relevant cell migration and proliferation [39, 40]. In under 4 passages, adult murine primary dermal fibroblasts (Cell Biologics, Chicago, IL, USA) were grown in 6-well plates to ~100% confluence using the manufacturer’s fibroblast medium (M2267). Media was removed, cells were rinsed with PBS, and then the monolayer was artificially wounded by scratching across each well with a 200 μl pipette tip (approximately 1 mm diameter). The wells were washed with fibroblast media to remove debris. Then, either fibroblast-conditioned fibroblast media or AT-84 cell-conditioned fibroblast media was applied (n = 6 wells/treatment). These conditioned media were generated from a 6-hour exposure to a ~75% confluent flask of the above-mentioned fibroblasts (control) or AT-84 cells (tumor cells used in *in vivo* experiments), respectively. At 0, 18, and 26 h post-scratching, images of the scratch in two areas of each well were photographed at 10X magnification using a CMOS digital camera mounted on the inverted microscope (Jenco, Portland, OR, USA) and accompanying ToupView software. Wound width was measured by a blind observer (H.L.), averaged for each well, and the percentage of scratch closure relative to the original scratch (0 h) was calculated and compared between media treatments.

### Statistical analysis

Differences in wound closure, tissue masses, mRNA expression, flow cytometry, and scratch width were analyzed using repeated measures or 2-way ANOVA with Statview version 5.0.1 software (Scientific Computing, Cary, NC, USA). Tumor treatment was treated as a between-subjects variable and time was treated as a within-subjects variable. Data were determined to be statistically significant when p < 0.05 and are presented as mean ± standard error of the mean (SEM).

## Results

### Body Weight

Relative to their weight on the day of tumor inoculation, body weight did not differ between tumor-bearing and–free mice on Day 1 post-wounding (change in body mass: control 2.4 ± 4.4, tumor-bearing 3.3 ± 2.6 g; p>0.05). Tumor mass increased from 0.18 ± 0.03 g at one day after wounding to 0.35 ± 0.03 g at 5 days post-wounding. As expected, spleen mass mirrored this increase in tumor mass for tumor-bearing mice from 0.12 ± 0.034 g at one day after wounding to 0.17 ± 0.04 g at 5 days post-wounding. As spleen mass did not change over time in tumor-free controls (Day 1: 0.1 ± 0.03 g; Day 5: 0.1 ± 0.02 g), spleen mass was significantly greater in tumor-bearing mice 5 days post-wounding relative to controls (t_1,8_ = 3.1; p<0.05).

### Cancer delays dermal wound closure

**Fig 1:**
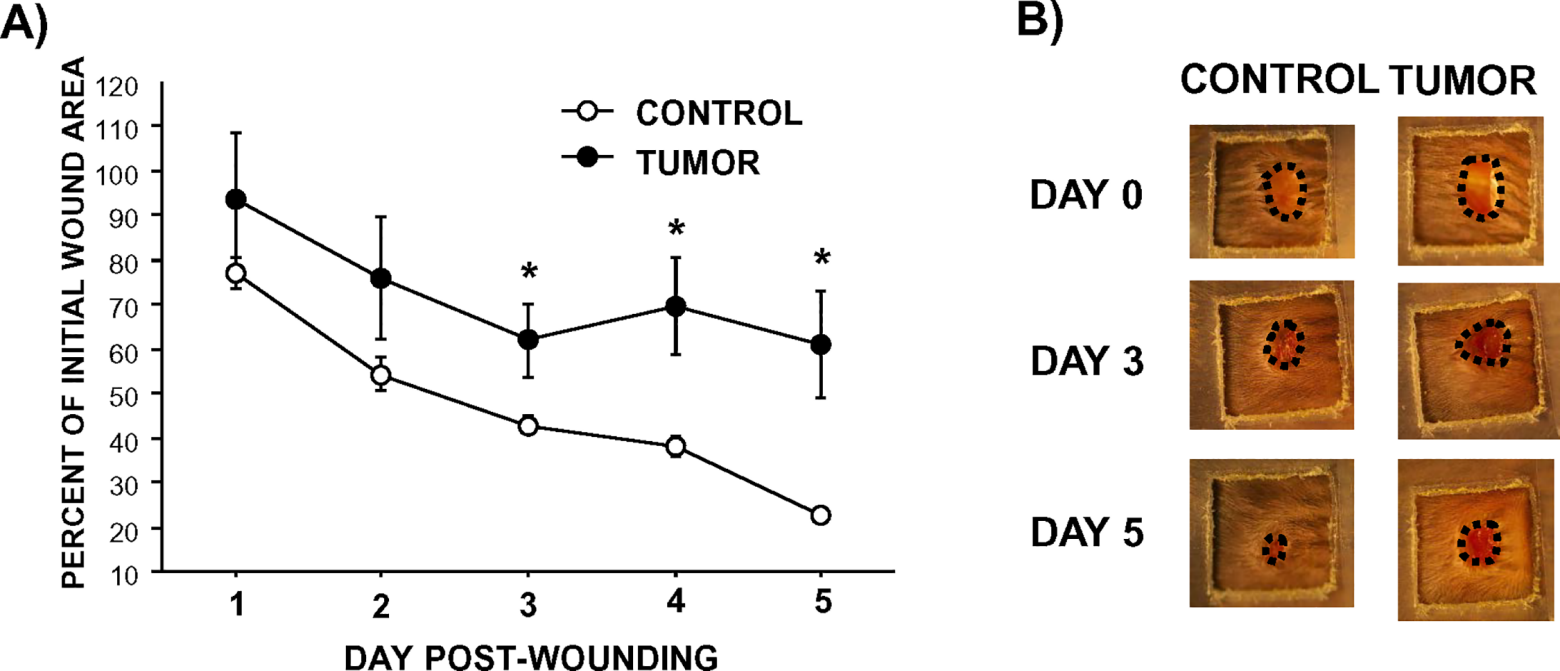
Tumors delay dermal wound closure. (A) Average percent (±SEM) of original 3.5 mm dermal excisional wound size analyzed over 5 days using digital photography. (B) Representative photographs of wounds over time. Unhealed skin is circled. n = 10/group; *p<0.05.

Dermal wound area, in all mice, decreased over the five days (F_4,72_ = 52.7, p<0.05). However, tumors reduced wound closure over time relative to tumor-free controls (F_4,72_ = 4.1, p<0.005; **Fig 1**).

### Wound myeloid cell infiltration

**Fig 2:**
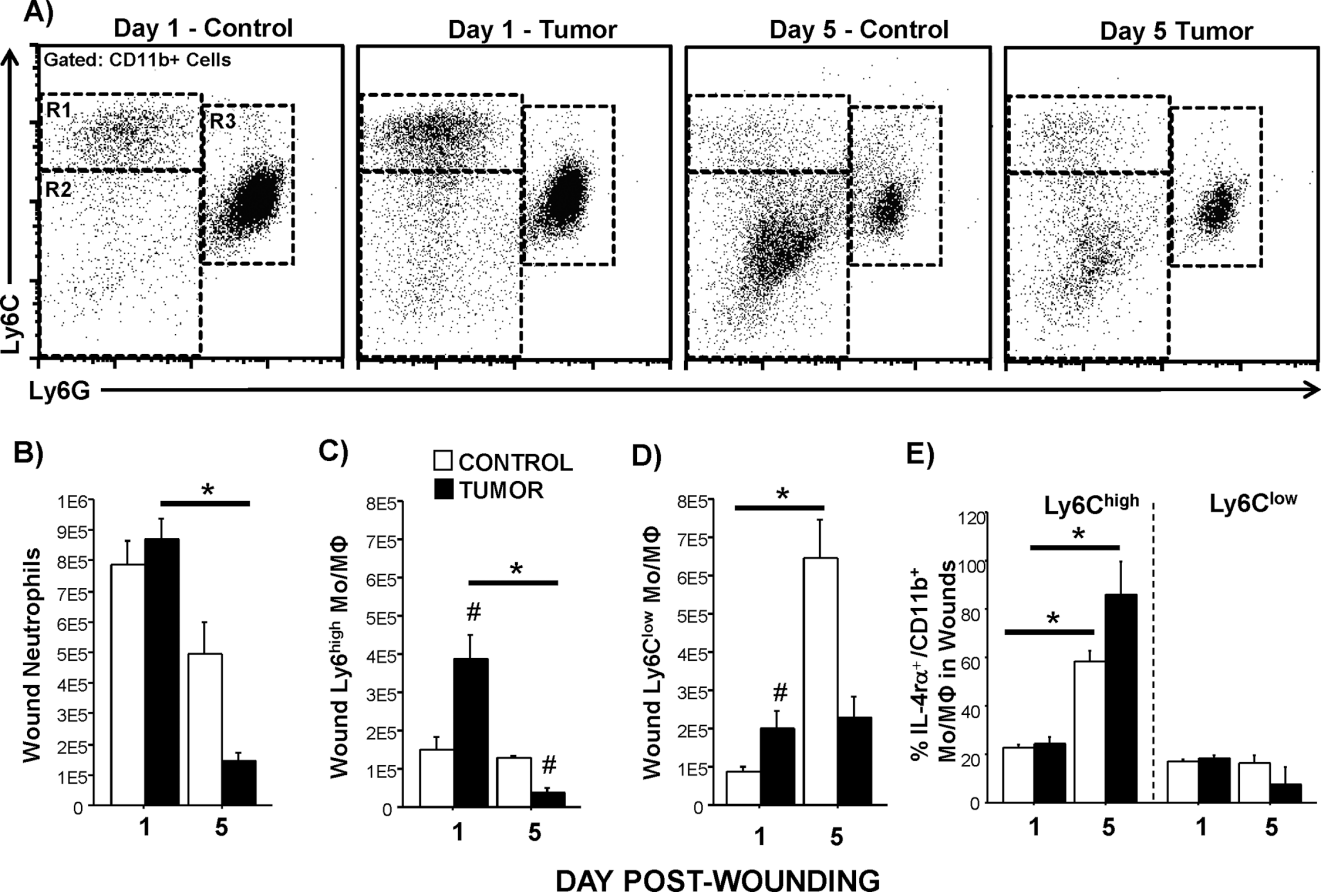
Tumors alter absolute myeloid cell numbers in wounds examined 1 or 5 days after wounding. Representative flow cytometry bivariate dot plots of Ly6C and Ly6G labeling of CD11b+ wound cells from mice (A) without tumors and with tumors 1 or 5 days post-wounding. Gates: R1 = Ly6C^high^ Mo/MΦ, R2 = Ly6C^low^Mo/MΦ, and R3 = Neutrophils. Average (±SEM) (B) neutrophil (Cd11b/Ly6G^+^), (C) Ly6C^high^ Mo/ MΦ, and (D) Ly6C^low^ Mo /MΦ isolated from wound tissues and quantified using flow cytometry. (E) Average (±SEM) percent of these Ly6C^high^ or Ly6C^low^ Mo/ MΦ expressing IL-4Rα protein. n = 2-3/group; *p<0.05 within the same cancer treatment over time; ^#^p<0.05 between treatments.

Neutrophils decreased in dermal wounds from Day 1 to Day 5 post-wounding in all mice, but only reached statistical significance in tumor-bearing mice (F_1,6_ = 4.4, p<0.05; **Fig 2A & 2B**). While tumor treatment interacted with time for wound neutrophils (F_1,6_ = 7.4, p<0.05), their Day 5 reduction with tumors did not reach statistical significance (p = 0.08).Total macrophage (Ly6C^high^ + Ly6C^low^) numbers rose in wounds from Day 1 to Day 5 post-wounding in controls as predicted, but tumors reversed this coordinated macrophage infiltration pattern (F_1,6_ = 24.4, p<0.01; data shown separately for Ly6C^high^ and Ly6C^low^; **Fig 2C & 2D**). Specifically, the number of mature macrophages (Ly6C^low^) increased over time in tumor-free mice as expected (**Fig 2D**). Conversely, although tumor-bearing mice displayed high counts of mature wound macrophages on Day 1 post-wound, the expected rise in mature cells by Day 5 was absent (F_1,6_ = 24.9, p<0.01; **Fig 2D**). Thus, in tumor-bearing mice, a lack of macrophage maturation occurred over time, despite these same mice having more immature (Ly6C^high^) wound macrophages with the potential to mature than controls (on Day 1; **Fig 2C**). Five days after wounding, numbers of immature macrophages in tumor-bearing mice dropped to levels even below those of tumor-free controls (F_1,6_ = 12.7, p<0.05, **Fig 2C**). Of these infiltrating wound Mo/MΦ, the expression of a myeloid-derived suppressor cell marker, IL-4Rα, increased in all mice (F_1,6_ = 6.8, p<0.05; **Fig 2E**), with a tendency for tumors to escalate this increase (p = 0.2). Taken together, myeloid cells entered (macrophages) and exited (neutrophils and macrophages) the wounds more rapidly in tumor-bearing mice than in tumor-free controls.

### Dermal wound qPCR

**Fig 3:**
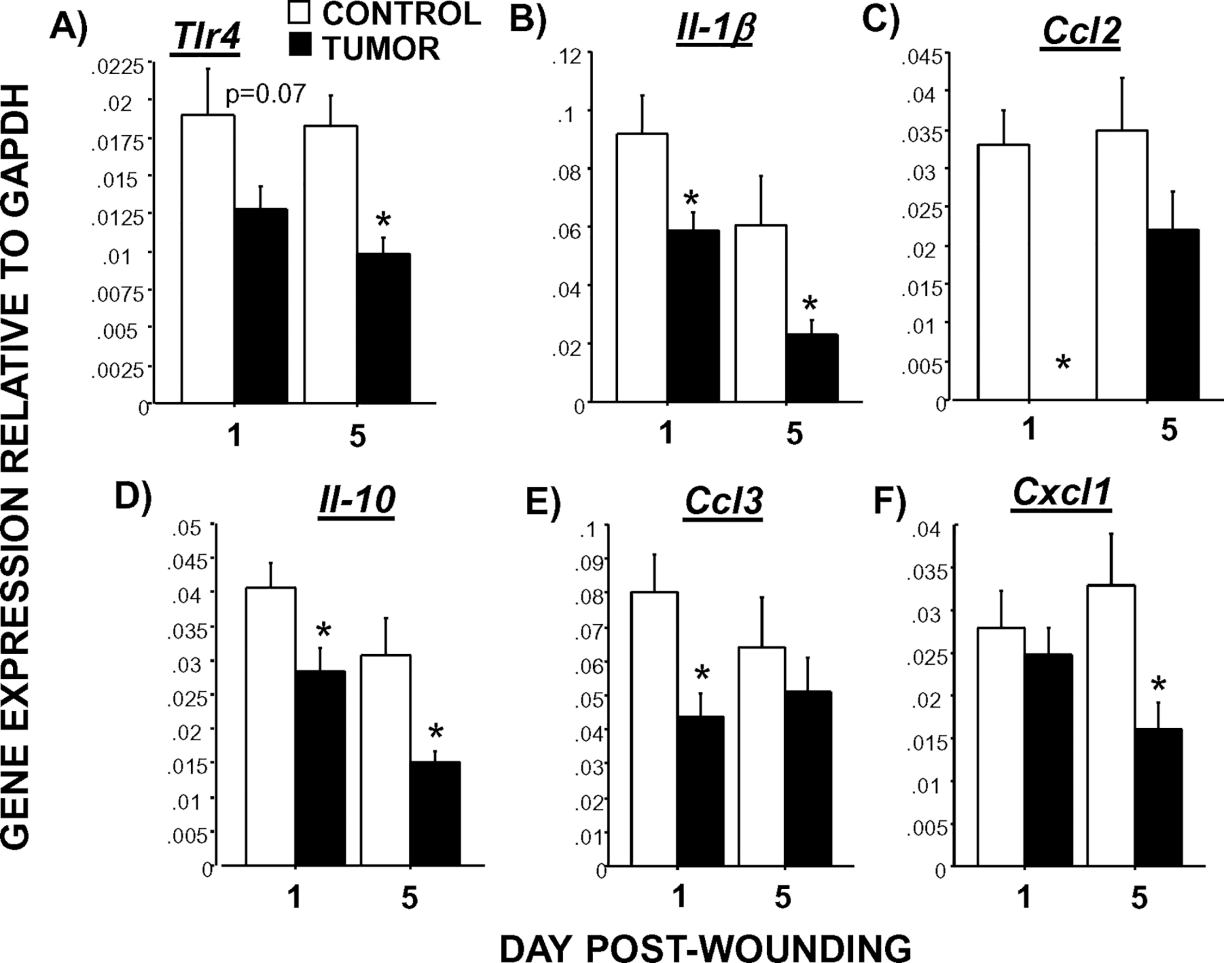
Tumors reduce relative gene expression of inflammatory markers in wounds. Average (±SEM) quantitative expression of (A) *Tlr4*, (B) *IL-1β*, (C) *Ccl2*, (D) *IL-10*, (E) *Ccl3*, and (F) *Cxcl1* mRNA extracted from wound tissue 1 or 5 days post-wounding. n = 8-10/group; *p<0.05 between cancer treatments.

**Fig 4:**
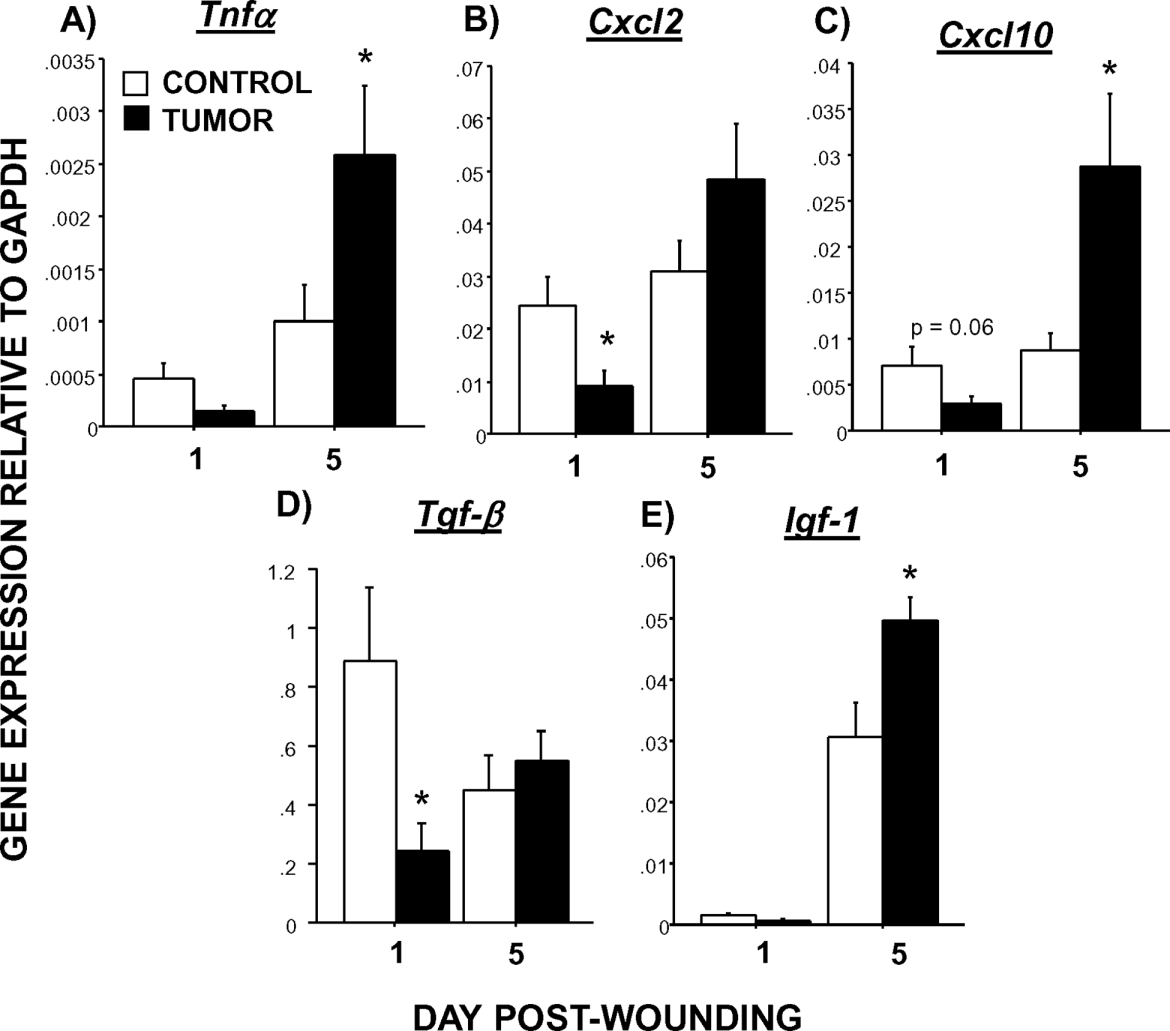
Tumors alter gene expression pattern of select inflammatory/growth factors in wounds over time. Average (±SEM) quantitative expression of (A) *Tnfα*, (B) *Cxcl2*, (C) *Cxcl10*, (D) *Tgf-β*, and (E) *Igf-1*mRNA extracted from wound tissue 1 or 5 days post-wounding. n = 8-10/group; *p<0.05 between cancer treatments.

The examination of inflammatory gene expression in wound tissues revealed two distinct and consistent patterns. First, tumors consistently reduced inflammatory gene expression; this was evident on Day 1 and/or Day 5 post-wounding, depending upon the marker (**Fig 3**). Statistically, this was represented by a significant main effect of tumor treatment (note: *Ccl2* also had a significant treatment x day interaction, F_1,36_ = 4.5, p<0.05). Tumors reduced *Il-1β* (F_1,36_ = 9.5, p<0.005), *Ccl3* (F_1,36_ = 5.1, p<0.05), *Cxcl1* (F_1,36_ = 5.4, p<0.05), *Il-10* (F_1,36_ = 13.7, p<0.005), and *Tlr4* (F_1,36_ = 13.4, p<0.005) in wounds. Secondly, a pattern of reduced inflammatory gene expression on Day 1 post-wounding followed by higher gene expression on Day 5 was observed in tumor-bearing mice relative to controls (**Fig 4**). This interaction of tumor treatment by time was statistically significant for *Tnfα* (F_1,36_ = 6.2, p<0.05), *Cxcl2* (F_1,36_ = 5.7, p <0.05), *Cxcl10* (F_1,36_ = 8.5, p<0.01), *Tgf-β* (F_1,33_ = 6.8, p<0.01), and *Igf-1* (F_1,36_ = 8.4, p<0.01). No statistically significant main effects of tumor or interactions with time were evident for other inflammation-related genes (*Il-6*, *Ccl5*, *Ccl1*, *Ccl22*, *Cx3cl1*; p>0.05).

### Circulating myeloid cell concentrations

**Fig 5:**
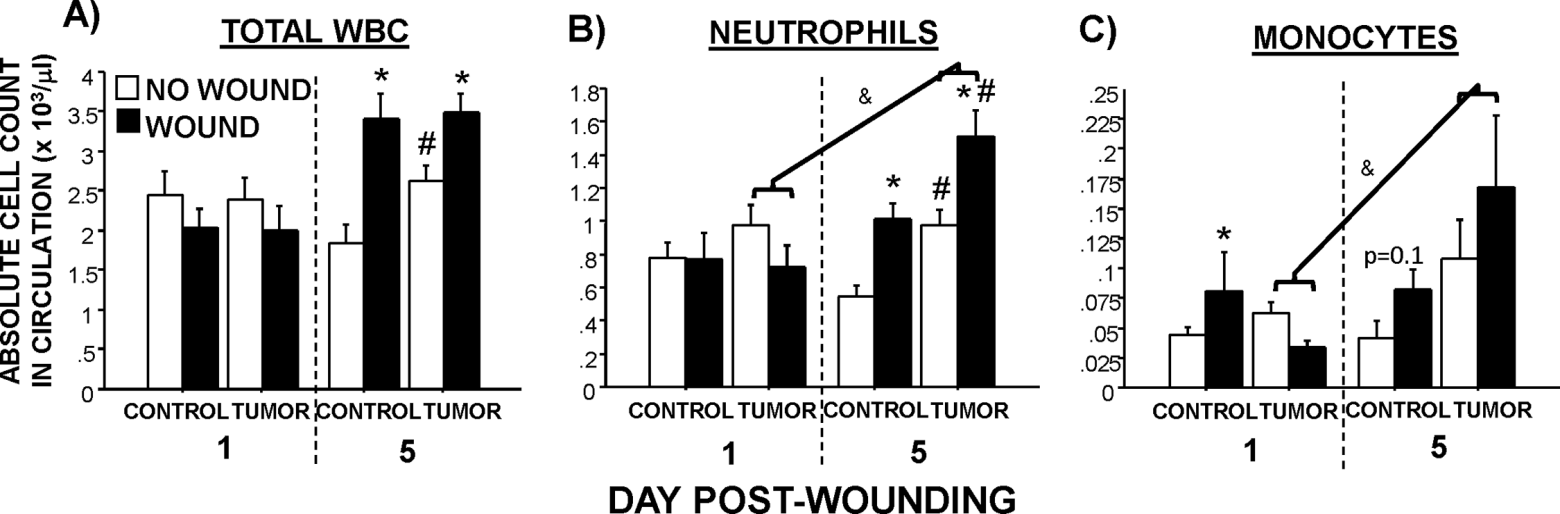
**Absolute circulating myeloid cell concentrations (CBC) with and without wounding.** Average (±SEM) circulating (A) total white blood cells, (B) neutrophils, and (C) monocytes in tumor-bearing and -free mice with and without dermal wounding. n = 8-10/group; *p<0.05 within the same cancer treatment between wounding treatments; #p<0.05 between cancer treatments with same wound treatment; &p<0.05 within the tumor groups between Days 1 & 5.

Tumors significantly elevated circulating neutrophils (F_1,52_ = 4.3, p<0.05) and monocytes (F_1,52_ = 6.1, p<0.05) over time, primarily driven by the 5 day post-wounding interval (**Fig 5**). Wounding further increased total white blood cell populations over time (F_1,52_ = 15.6, p<0.01), and specifically, neutrophils (F_1,52_ = 10.7, p<0.01). Wounding tended to modulate circulating monocyte increases in tumor-bearing mice one day post-wounding (F_1,33_ = 3.8, p = 0.06).

### Scratch Assay

**Fig 6:**
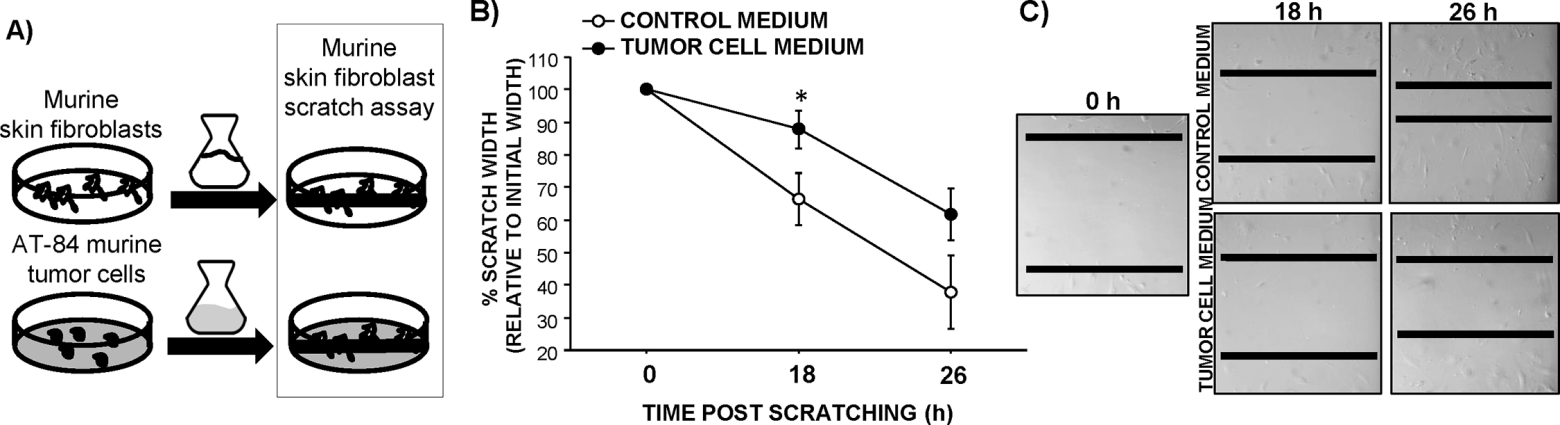
Tumor cell-conditioned media reduces murine fibroblast migration and proliferation. (A) Schematic of conditioned media paradigm and in vitro “wounding” scratch assay. (B) Average (±SEM) percent decrease in scratch “wound” width of adult murine fibroblasts 18 and 26 h post-scratching cultured with fibroblast-conditioned control media or tumor cell-conditioned media. n = 6 wells/group; *p≤0.05 between treatments (C) Representative photographs of scratch assay. Black bars represent margins of scratch.

Migration and proliferation of adult murine fibroblasts reduced the scratch width over time in all wells (F_1,10_ = 27.1; p<0.01; **Fig 6**). However, tumor cell-conditioned media attenuated the scratch recovery or “healing” relative to fibroblast-conditioned control media at 18 h post-scratching (p≤0.05) with a similar trend at 26 h post-scratching (p = 0.1).

## Discussion

The overall reduction in wound closure in tumor-bearing mice (and *in vitro*) may represent a delay in closure or the manifestation of a non-healing wound phenotype. While previous studies indicate that healed wound tissue may be weaker in tumor-bearing rats [9–11], this is the first study to empirically assess wound closure kinetics in the context of cancer. The rate of wound closure is a measure of the early and tightly-coordinated inflammatory and proliferative phases of wound healing. It represents the window of time in which the body is particularly vulnerable to external pathogens. Therefore, delayed wound closure increases the risk of potential bacterial infection, which in turn, can prolong pain and increase hospital stay and cost [41]. Indeed, bacterial infection incidence is higher in tumor resection surgeries compared with elective surgery [41]. Given that the trajectory of wound closure in the tumor-bearing mice appears to be flattening out, further studies are necessary to determine the extent to which this closure delay persists. Of note, this tumor model did not result in cachexia (e.g., weight loss), therefore, the slower healing kinetics were not due to overall metabolic changes. This work suggests that tumor biology itself is sufficient to impair tissue repair, which is likely to be compounded by chemotherapy, radiation, and other cancer-treatments [42–44].

Differences in wound closure between groups became apparent relatively early in the inflammatory phase (Day 2–3), suggesting that leaking, infiltration, recruitment, or function of myeloid cells into the wound were altered. Indeed, this was strongly supported by the wound flow cytometry data. Both immature (Ly6C^high^) and mature Mo/MΦ rapidly infiltrated (or were already resident) into the wounds of tumor-bearing mice one day after wounding. This was faster than expected in a dermal excisional wound model, which classically has the peak macrophage infiltration closer to 3–5 days post-wounding [29, 45] (see tumor-free control data). This may be supported by the finding that smaller percentages of macrophages drained out of tumor-resected wounds in a clinical study than from wounds of non-cancerous surgeries [25]. This rapid infiltration may reflect a general increase in bone marrow myelopoiesis [46] and rapid shunting of monocytes in the circulation to wounds in the tumor model, both of which are supported by the circulating myeloid cell results. Wounding tended to decrease circulating monocytes on Day 1 in the tumor-bearing mice, but increased them in controls. In fact, some of these infiltrating cells may be comparable to myeloid-derived suppressor cells in tumors given their tendency to express IL-4Rα [47]. Potential functional suppression of immune activity by these cells requires further investigation. However, 1) mature macrophages, not just bone-marrow derived immature monocytes, were elevated in the wounds from tumor-bearing mice, and 2) neutrophils did not appear to mirror this early over-abundance. The distinct possibility that macrophages were previously trafficked to unwounded skin during tumor growth is under current investigation. Of note, the roles of neutrophils and macrophages in wound healing are somewhat debated. While these cells release many cytokines and growth factors and protect and clean the wound [48, 49], they may not be absolutely necessary for tissue repair as demonstrated by studies of fetal wound healing and using myeloid cell-depleted knock-out newborn mice [50, 51]. Further, in models of other chronic diseases that are characterized by delayed wound healing (e.g., diabetes), macrophage and/or neutrophil depletion has unexpectedly improved healing [52, 53]. This suggests that the early abundance of macrophages in the wounds of tumor-bearing mice may, in fact, be detrimental to wound closure. Previous work from our lab indicates that wound myeloid cell flow cytometry in other models corroborates both wound histological analyses of myeloid cell infiltration and gene expression analyses [54].

In contrast, both neutrophils and Mo/MΦ were reduced in wounds of tumor-bearing mice later in the healing process (5 days post-wounding). At this time, neutrophils are typically cleared out of the wounds through apoptosis and phagocytosis, however, here the decline in wound neutrophils was greater in tumor-bearing mice than controls. Likewise, for Mo/MΦ, the immature Ly6C^high^ Mo/MΦ not only decreased over time, but fell significantly below that of tumor-free mice on Day 5. Concurrently, circulating monocytes and neutrophils were elevated in the tumor-bearing mice. This suggests that monocytes from the bone marrow were either recruited to the wounds less in tumor-bearing mice (as some wound chemokine and chemotactic data suggested) at this later phase of healing and/or emigrated out of the wounds via the lymphatic system more than tumor-free mice. It is unlikely that these immature cells simply turned over into a more mature phenotype, as there was no increase in mature Mo/MΦ at this time. This maturation process is evident in the controls, however, as phenotypically “mature” Mo/MΦ were significantly elevated by Day 5 post-wounding. These Ly6C^low^ Mo/MΦ are hypothesized to differentiate into pro-healing macrophages [55]. Taken together, the initial elevation in Mo/MΦ and the later decline in both neutrophils and Mo/MΦ in wounds of tumor-bearing mice suggest that the wound microenvironment may be uninhabitable to these myeloid cells [56] or that these cells were quickly drawn out, possibly by the competing needs of the distal tumor. Indeed, while the present research focuses on how tumors affect wounds, a robust body of research from the Ben-Eliyahu laboratory has demonstrated that surgery promotes metastasis [57]. Based on this work, which demonstrates the negative effects of endocrine responses to surgery (due to stress and tissue damage) on metastases, future studies examining the role of catecholamines in the present model are warranted. Taken together, the physiological interactions between wounds and cancer are likely bi-directional.

Six of the twelve healing-related inflammatory genes found to be affected were consistently reduced in wounds of tumor-bearing mice relative to tumor-free controls. *Tlr4* has been shown to increase early in excisional wound repair and is an upstream component of signaling pathways leading to local cytokine production (e.g., IL-1β) [58]. Congruent with this pathway, wound *Tlr4* mRNA was reduced in tumor-bearing mice, along with downstream *IL-1β* and *Ccl2*. This transcript immunosuppression in tumor-bearing mouse wounds is consistent with the early elevations in wound Mo/MΦ being designated as MDSCs. The lack of pro-inflammatory signaling may have also contributed to the later Mo/MΦ emigration [59] in tumor-bearing mouse wounds. In addition, expression of factors that initiate the resolution of inflammation (*IL-10*, *Tgf-β*) were also diminished one day after wounding in wound tissues of tumor-bearing mice relative to controls. Reduction in the neutrophil chemokine, *Cxcl1*, corroborated the relative decrease in wound neutrophils (Day 5 post-wounding) of tumor-bearing mice. In contrast, the macrophage chemokines, *Ccl2* and *Ccl3*, were lower in wounds of mice with tumors, while wound Mo/MΦ were high. This again may indicate that some of the Mo/MΦ localization was due to leaking from blood vessels upon tissue disruption rather than active recruitment of Mo/MΦ via signaling. Reduced Mo/MΦ chemotactic signals may also be related to the quickly reduced numbers of wound Mo/MΦ by Day 5 post-wounding. Of note, previous work in other models in our lab indicate that inflammatory signal mRNA in wound tissue is comparable to that of protein, suggesting that these gene expression data are biologically relevant [54].

Six out of twelve of the early healing genes found to be altered displayed a pattern of early reduction and later elevation in wounds of tumor-bearing mice relative to controls. Given that this pattern is opposite to the Mo/MΦ pattern, the two may be inversely related. Of note, these expression profiles were observed in two neutrophil chemokines, *Cxcl2* and *Cxcl10*, and one of the macrophage chemokines examined (fractalkine, *Cx3cl1*). Delayed elevations in wound *Cxcl2* are also observed in wounds of chronically stressed mice [54], in which wound macrophage numbers are not altered, but their phagocytic activity is diminished. The late elevations in the wound inflammatory markers (*Tnfα*, chemokines) specifically indicate a delay or dysregulation of the inflammatory phase (Days 1–3 post-wounding). In summary, these gene expression analyses suggest that proper immune cell recruitment and both the initiation and resolution of the ensuing wound inflammatory response are impaired in the presence of a tumor. Of note, these inflammatory markers may represent signals coming from not only immune cells in the wounds, but also platelets, mast cells, and fibroblasts [27, 60]. As this initial study used whole wound samples, future studies can be directed to isolate and sort out specific immune cells from the wounds and thereby differentiate the specific transcript (and protein) profiles of various cell types. However, neither of these distinct patterns of gene expression explains the initial increase in dermal wound monocytes in tumor-bearing mice. The possibility that monocyte trafficking to the skin may already be elevated prior to wounding due to the presence of a tumor is a likely hypothesis that we are pursuing [61].

Based on the *in vitro* “wounding” assay, we conclude that humoral signals from the tumor cells can directly affect migration and/or proliferation of fibroblast cells. Because solid tumors consist of various cell types (tumor cells, immune cells, endothelial cells, fibroblasts), this is a valid distinction. In our model, the tumors are approximately 15 mm upstream from wound site (unpublished data) in the direction of the venous circulatory return, making direct humoral effects of tumor factors on skin biology a possibility. Combined with the *in vivo* work, these data indicate that tumors may secrete factors which alter healing through both direct (e.g., tumor cell growth factor or cytokine secretion) and indirect (e.g., myeloid cell actions) mechanisms of action. Furthermore, these data suggest that in addition to the inflammatory phase, the later proliferative phase may be independently affected by tumors.

Taken together, the delayed or suppressed inflammatory transcriptional findings paired with those of altered immune cell counts from tumor-bearing mice corresponded with the observed delays in wound closure. There is evidence that head and neck cancer alters humoral immunity in cancer survivors long after successful treatment [62, 63]. The present model has the potential to explore the same persistence of cancer-induced alterations in innate immunity. This research indicates that fundamental immune processes are altered by tumor biology alone and suggests that interventions targeting these clinically-relevant immune functions may significantly improve patient quality-of-life and augment treatment and mortality outcomes.
